# Supplementary material for: Fatty acid-binding protein 4 is an independent factor in the pathogenesis of retinal vein occlusion
Source: PLoS One. 2021 Jan 27;16(1):e0245763. doi: 10.1371/journal.pone.0245763 (PMC7840053; doi:10.1371/journal.pone.0245763)
Supplement: S1 Table — Variables are expressed as number, means ± SD or medians (interquartile ranges). AST, aspartate transaminase; ALT, alanine transaminase; eGFR, estimated glomerular filtration rate; γGTP, γ-glutamyl transpeptidase; hsCRP, high-sensitivity C-reactive protein; MA, mean blur rate (MBR) of all area of optic verve head (ONH); MV, MBR of the vascular area of ONH; MT, MBR of the tissue area of ONH. * P < 0.05 vs. non-RVO. † P < 0.05 vs. BRVO. (DOCX) [file pone.0245763.s001.docx]

S1 Table. Characteristics of the patients with non-RVO, BRVO and CRVO (n = 32)

|  |  | All | non-RVO | BRVO | CRVO | P |
| --- | --- | --- | --- | --- | --- | --- |
| n |  | 32 | 18 | 9 | 5 |  |
| Sex (Male/Female) | | 12/20 | 6/12 | 4/5 | 2/3 | 0.847 |
| Age (years) | | 68 ± 11 | 68 ± 8 | 69 ± 13 | 65 ± 19 | 0.701 |
| Body mass index | | 23.8 ± 3.0 | 23.2 ± 3.4 | 25.5 ± 2.2 | 23.1 ± 1.9 | 0.136 |
| Systolic blood presure (mmHg) | | 137 ± 16 | 137 ± 17 | 138 ± 17 | 137 ± 17 | 0.945 |
| Diastolic blood pressure (mmHg) | | 80 ± 10 | 80 ± 10 | 83 ± 13 | 76 ± 8 | 0.569 |
| Biochemical data | |  |  |  |  |  |
|  | Total choleterol (mg/dL) | 204 ± 33 | 208 ± 40 | 196 ± 22 | 202 ± 18 | 0.564 |
|  | Triglycerides (mg/dL) | 155 (103-220) | 120 (96-222) | 192 (94-235) | 170 ± 34 | 0.598 |
|  | Fasting glucose (mg/dL) | 111 (99-134) | 115 (101-147) | 121 (93-130) | 104 (89-110) | 0.158 |
|  | Hemoglobin A1c (%) | 6.0 ± 0.7 | 6.1 ± 0.9 | 6.0 ± 0.5 | 6.0 ± 0.4 | 0.929 |
|  | Blood urea nitrogen (mg/dL) | 15 ± 5 | 15 ± 4 | 13 ± 4 | 16 ± 7 | 0.539 |
|  | Creatinine (mg/dL) | 0.7 (0.6-0.8) | 0.7 (0.6-0.8) | 0.8 (0.6-0.9) | 0.7 (0.6-0.8) | 0.671 |
|  | eGFR (mL/min/1.73m^2^) | 71.7 ± 1.2 | 71.0 ± 17.4 | 71.9 ± 15.8 | 73.7 ± 13.7 | 0.935 |
|  | Uric acid (mg/dL) | 5.0 ± 1.2 | 5.3 ± 1.2 | 4.6 ± 0.8 | 4.9 ± 2.0 | 0.337 |
|  | AST (IU/L) | 24 (19-31) | 26 (20-33) | 24 (17-31) | 19 (16-28) | 0.157 |
|  | ALT (IU/L) | 23 (15-28) | 24 (16-29) | 22 (14-32) | 16 (13-47) | 0.763 |
|  | γGTP (IU/L) | 29 (16-53) | 26 (15-61) | 33 (18-52) | 38 (20-63) | 0.682 |
|  | hsCRP (mg/dL) | 0.09 (0.04-0.13) | 0.06 (0.03-0.12) | 0.10 (0.04-0.13) | 0.10 (0.06-0.26) | 0.493 |
| Laser speckle flowgraphy | | [n = 27] | [n = 18] | [n = 6] | [n = 3] |  |
|  | MA | 19.3 ± 6.0 | 21.0 ± 5.8 | 17.2 ± 3.7 | **12.7 ± 5.9*** | **0.045** |
|  | MV | 33.9 ± 9.4 | 36.0 ± 7.4 | 34.7 ± 10.1 | **19.7 ± 9.1*†** | **0.014** |
|  | MT | 12.0 ± 3.3. | 12.7 ± 3.3 | 10.8 ± 3.1 | 10.2 ± 3.9 | 0.305 |
|  | MV-MT | 21.9 ± 7.2 | 23.3 ± 5.6 | 23.9 ± 7.1 | **9.5 ± 5.3*** | **0.003** |
|  | MM | 8.5 ± 4.4 | 9.1 ± 4.8 | 8.4 ± 2.5 | 4.5 ± 3.6 | 0.250 |

Variables are expressed as number, means ± SD or medians (interquartile ranges).

AST, aspartate transaminase; ALT, alanine transaminase; eGFR, estimated glomerular filtration rate; γGTP, γ-glutamyl transpeptidase; hsCRP, high-sensitivity C-reactive protein; MA, mean blur rate (MBR) of all area of optic verve head (ONH); MV, MBR of the vascular area of ONH; MT, MBR of the tissue area of ONH.

* P < 0.05 vs. non-RVO. † P < 0.05 vs. BRVO
