# Supplementary material for: Fatty acid-binding protein 4 is an independent factor in the pathogenesis of retinal vein occlusion
Source: PLoS One. 2021 Jan 27;16(1):e0245763. doi: 10.1371/journal.pone.0245763 (PMC7840053; doi:10.1371/journal.pone.0245763)
Supplement: Attachment — Submitted filename: Reply_PLOSONE.docx [file pone.0245763.s002.docx]

Dear Editor,

This is to thank you very much for the constructive comments on our revised manuscript entitled “*Fatty acid-binding protein 4 is an independent factor in the pathogenesis of retinal vein occlusion.*” I carefully checked all of comments from the reviewers and have made specific changes to our manuscript as follows;

**Reviewer 's Comments:**

**Reviewer #1:**

1) *I recommend adding the following references to line 5 Pag. 4:*

*…… agents substantially improved ME and visual acuity (VA) (7*

*Pacella F, La Torre G, Basili S, et al. Comparison between "early" or "late" intravitreal injection of dexamethasone implant in branch (BRVO) or central (CRVO) retinal vein occlusion: six-months follow-up. Cutan Ocul Toxicol. 2017 Sep;36(3):224-230. doi: 10.1080/15569527.2016.1254648. Epub 2017 Jan 11. PMID: 27903073.*

line 7 Pag. 4:

*………. even though VA and ME are improved (10*

*Pacella E, Pacella F, La Torre G, et al.* *Testing the effectiveness of intravitreal ranibizumab during 12 months of follow-up in venous occlusion treatment. Clin Ter. 2012 Nov;163(6):e413-22. PMID: 23306756.*

**Answer;** As pointed out, these recommended references are now included in the paper.

2) I recommend adding the following references to line 10 Pag. 4:

………. risk factor for RVO (1,

*Romiti GF, Corica B, Borgi M, Visioli G, Pacella E, Cangemi R, Proietti M, Basili S, Raparelli V.* *Inherited and acquired thrombophilia in adults with retinal vascular occlusion: A systematic review and meta-analysis. J Thromb Haemost. 2020 Aug 17. doi: 10.1111/jth.15068. Epub ahead of print. PMID: 32805772*.

*Pacella F, Bongiovanni G, Malvasi M, Trovato Battagliola E, Pistone A, Scalinci SZ, Basili S, La Torre G, Pacella E.* *Impact of cardiovascular risk factors on incidence and severity of Retinal Vein Occlusion. Clin Ter. 2020 Nov-Dec;171(6):e534-e538. doi: 10.7417/CT.2020.2269. PMID: 33151253.*

*Stefanutti C, Mesce D, Pacella F, et al.* *Optical coherence tomography of retinal and choroidal layers in patients with familial hypercholesterolaemia treated with lipoprotein apheresis. Atheroscler Suppl. 2019 Dec;40:49-54. doi: 10.1016/j.atherosclerosissup.2019.08.031. PMID: 31818450.*

*Bianchi E, Scarinci F, Ripandelli G, Feher J, et al.* *Retinal pigment epithelium, age-related macular degeneration and neurotrophic keratouveitis. Int J Mol Med. 2013 Jan;31(1):232-42. doi: 10.3892/ijmm.2012.1164. Epub 2012 Oct 26. PMID: 23128960.*

**Answer;** As pointed out, these recommended references are now included.

**Reviewer #2:**

1) While on the whole the paper is readable, some grammar should be checked and rewritten, and professional English language editing is required.

**Answer;** As pointed out, the paper has been revised by a native English editing service.

2)Results

Please pay attention to number, grammar and spelling mistakes, such as patients with RVO (n=20), GTP. This sentence” and ALT, GTP and hsCRP, and LSFG ocular blood flow indexes including MA” was showed as a picture format, which should be revised. In Table 1, the MT data should be checked.

**Answer;** As pointed, I confirmed and changed a picture format. In addition, the MT data in Table 1 has been corrected.

3)Discussion

I would suggest rewriting the discussion section, especially second half of first paragraph which should not only repeat the results section. In addition, correlation between FABP4 and LSFG mean blur rate (MBR) values of ONH should be more discussed.

**Answer;** As pointed, I deleted the second half of first paragraph that included the repeating of the results section within Discussion 1^st^ paragraph as follows; “Upon retinal hypoxia, VEGF is expressed in various cells within the retina including retinal glial cells, retinal pigment epithelial cells, and vascular endothelial cells (40), and in turn, VEGF increases vascular permeability and promotes the proliferation of endothelial cells (41, 42). In terms of the intraocular VEGF level, it was reported that those levels are not necessarily elevated in all RVO patients (43), and are widely varied among patients. In fact, it was reported that the intraocular VEGF level was significantly correlated with the retinal non-perfused area (44), as well as the severity of macular edema (43). Based upon these findings, it would appear that the expression of VEGF initially increases after RVO due to retinal hypoxia caused by a vascular occlusion, leading to the disruption of the BRB and the development and progression of macular edema. Several previous studies have demonstrated that the velocity of retinal blood flow is lower in RVO patients than in subjects with normal eyes (45-47), and this reduction in blood flow velocity was correlated with the severity of RVO (48, 49). In the present study, we also found a significant increase of V-VEGFA and a decrease in LSFG ocular blood flow at ONH (MA) in patients with RVO, and those changes were more evident in CRVO as compared to BRVO. In addition, based on the findings of this study, we conclude that FABP4 is an exclusively independent factor, and the possibility that it is synergistically involved in the pathogenesis of RVO with VEGFA cannot be excluded.”.

In terms of correlation between FABP4 and LSFG mean blur rate (MBR) values of ONH, the corresponding discussion is included in the Discussion 2^nd^ paragraph as follows; “It has been reported that the FABP4 is considered to be primarily an adipocyte- and macrophage-specific protein, and plays an important role in maintaining glucose and lipid homeostasis (25, 27). While the issue of why vitreous specimens obtained from patients with RVO have such high concentrations of adipocyte- and macrophage-specific FABP4 remains unclear, recent studies suggest that FABP4 is more widely expressed in a wide variety of tissues including capillaries and veins ( but not arteries) and endothelial cells under normal conditions (50). *Therefore, these findings suggest that the V-FABP4 is most likely derived from retinal capillaries and venous tissue that is affected by RVO, and we therefore conclude that V-FABP4 may be pivotally involved in the regulation of the ocular blood circulation. In fact, the LSFG index of MV-MT at post-operative 1week was independently associated with Log V-FABP4 but not with Log V-VEGFA in our stepwise multivariable regression analyses. In support of this, in another study, it was reported that the velocity of retinal blood flow by LSFG was more strongly correlated with inflammatory factors than VEGF in patients with nonischemic CRVO and macular edema (51).*”.
